# Supplementary figures and images for: Genomic heritability estimates in sweet cherry reveal non-additive genetic variance is relevant for industry-prioritized traits
Source: BMC Genet. 2018 Apr 10;19:23. doi: 10.1186/s12863-018-0609-8 (PMC5894190; doi:10.1186/s12863-018-0609-8)

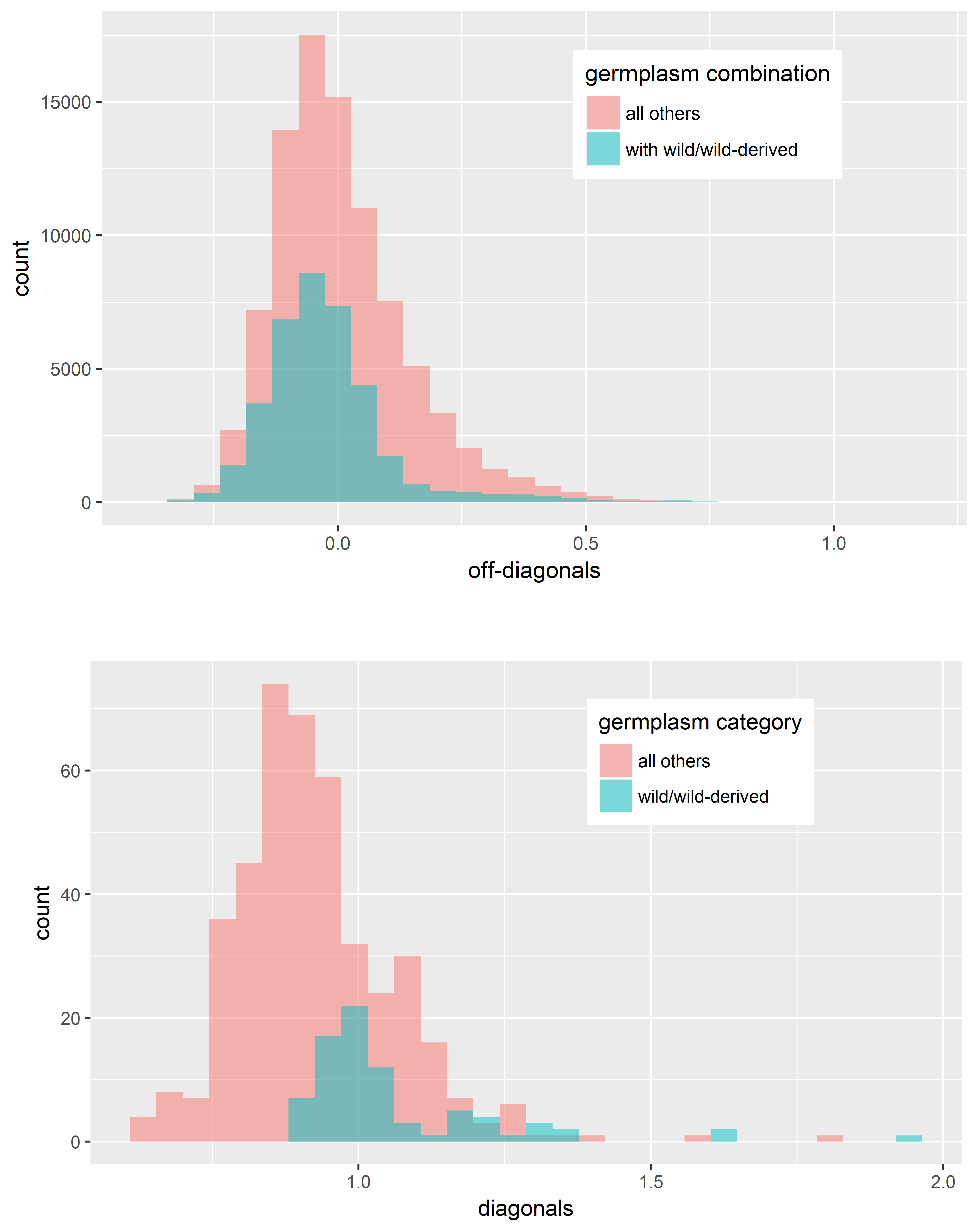

Supplement: Supplementary file 3 — Histogram of the diagonals and off-diagonals from the additive relationship matrix. (PNG 268 kb) [file 12863_2018_609_MOESM3_ESM.png]
